# Orbitofrontal sulcal patterns in catatonia

**DOI:** 10.1192/j.eurpsy.2023.2461

**Published:** 2023-10-19

**Authors:** Mylène Moyal, Alexandre Haroche, David Attali, Ghita Dadi, Matthieu Raoelison, Alice Le Berre, Anton Iftimovici, Boris Chaumette, Sylvain Leroy, Sylvain Charron, Clément Debacker, Catherine Oppenheim, Arnaud Cachia, Marion Plaze

**Affiliations:** 1 GHU Paris Psychiatrie et Neurosciences, Hôpital Sainte Anne, Paris, France; 2 Université Paris Cité, Institute of Psychiatry and Neuroscience of Paris (IPNP), INSERM U1266, IMA-Brain, Paris, France; 3 Physics for Medicine Paris, Inserm U1273, CNRS UMR 8063, ESPCI Paris, PSL University, Paris, France; 4 Université Paris Cité, Laboratory for the Psychology of Child Development and Education, CNRS UMR 8240, Sorbonne, Paris, France; 5 NeuroSpin, Atomic Energy Commission, Gif sur Yvette, France; 6 Department of Psychiatry, McGill University, Montreal, QC, Canada

**Keywords:** catatonic syndrome, neurodevelopment disorder, prefrontal cortex, schizophrenia, sulcogyral patterns

## Abstract

**Background:**

Catatonia is a psychomotor syndrome frequently observed in disorders with neurodevelopmental impairments, including psychiatric disorders such as schizophrenia. The orbitofrontal cortex (OFC) has been repeatedly associated with catatonia. It presents with an important interindividual morphological variability, with three distinct H-shaped sulcal patterns, types I, II, and III, based on the continuity of the medial and lateral orbital sulci. Types II and III have been identified as neurodevelopmental risk factors for schizophrenia. The sulcal pattern of the OFC has never been investigated in catatonia despite the role of the OFC in the pathophysiology and the neurodevelopmental component of catatonia.

**Methods:**

In this context, we performed a retrospective analysis of the OFC sulcal pattern in carefully selected homogeneous and matched subgroups of schizophrenia patients with catatonia (*N* = 58) or without catatonia (*N* = 65), and healthy controls (*N* = 82).

**Results:**

Logistic regression analyses revealed a group effect on OFC sulcal pattern in the left (*χ* ^2^ = 18.1; *p* < .001) and right (*χ* ^2^ = 28.3; *p* < .001) hemispheres. Catatonia patients were found to have more type III and less type I in both hemispheres compared to healthy controls and more type III on the left hemisphere compared to schizophrenia patients without catatonia.

**Conclusion:**

Because the sulcal patterns are indirect markers of early brain development, our findings support a neurodevelopmental origin of catatonia and may shed light on the pathophysiology of this syndrome.

## Introduction

Catatonia is a psychomotor syndrome frequently observed in disorders with neurodevelopmental impairments, including psychiatric disorders such as schizophrenia, unipolar and bipolar disorders, and autism spectrum disorder (ASD) [1, 2]. Catatonia is characterized by a major default in the interaction between the patient and his environment with an impairment in the ability to initiate or terminate movement, speech, and emotion despite the integrity of physical abilities [3].

The orbitofrontal cortex (OFC), a brain area involved in social abilities, emotional regulation, sensory-visceral-motor integration, motivation, decision-making, and goal-directed behavior [4–6] likely plays a key role in the pathophysiology of catatonia [7, 8]. Indeed, the OFC has been repeatedly associated with catatonia, including GABAergic deficits during negative emotional processing and poor connectivity with medial prefrontal cortices [9–13] along with reduced local cortical surface area and increased local gyrification index [14]. The critical role of the OFC in decision-making and goal-directed behavior is particularly important in the context of catatonia. Indeed, individuals with catatonia exhibit episodes of purposeless agitation in addition to periods of immobility along with an inability to execute basic movements [8]. The involvement of the OFC in emotional processing is also relevant in catatonia, as it may be intrinsically linked to emotional overload [15].

Complementary to these local features of the OFC, the OFC also present with an important interindividual gross morphological variability [16], with three distinct H-shaped sulcal patterns – referred as Types I, II, or III – based on the continuity of the medial and lateral orbital sulci [17]. The sulcal pattern of the OFC is considered a neurodevelopmental vulnerability marker for psychiatric disorders, and particularly for schizophrenia [18–27]. Several studies have reported less type I and more type II and type III in schizophrenia patients compared to healthy controls [24]. In schizophrenia patients, the type III is also associated with more severe symptoms, poorer cognitive function, more impulsivity, and earlier age of onset [23, 27]. In recent years, there has been increased recognition of catatonia as a neurodevelopmental disorder [2, 28], supporting neurodevelopmental deviations as key risk factors for the emergence of catatonia in both pediatric and adults populations [1, 29]. Therefore, studying the neurodevelopmental predisposition to catatonia by assessing sulci patterns is essential to test these hypotheses.

The sulcal pattern of the OFC has never been investigated in catatonia despite the role of the OFC in the pathophysiology of catatonia and the increasingly documented neurodevelopmental component of catatonia [2, 29]. In this context, the aim of the present study was to analyze the sulcal pattern of the OFC in carefully selected homogeneous and matched subgroups of schizophrenia patients with or without catatonia. We anticipated more type III sulcal patterns in schizophrenia patients with catatonia since catatonia can be considered a clinical complication of schizophrenia [30] and type III is associated with severe forms of schizophrenia [23, 27].

## Methods

### Participants

The number of participants to be included in this retrospective study was based on a previous study reporting the difference in sulcal pattern types between schizophrenia patients and healthy controls [25]. With alpha error set at .05, a power at 90% and one-sided tests, the sample size calculation indicated a total of 116 subjects. Of note, this is the brain imaging study of catatonia with the largest sample size.

In order to identify potential catatonia patients in our psychiatry ward, we retrospectively searched for all inpatients in the university hospital department at Sainte-Anne Hospital, Paris, France, between January 2015 and March 2022 with lorazepam prescriptions, a standard and specific treatment for catatonia [31, 32]. For each prescription of lorazepam, the following words were searched in the hospital report and the computerized record: “catatonia,” “catatonic syndrome,” “Bush Francis,” “BFCRS” (the Bush and Francis Catatonia Rating Scale, BFCRS, is the current gold standard scale for assessing catatonia and its severity) [33]. A patient was further considered in the study if the diagnosis of catatonia was confirmed by the clinicians who took care of the patient during the hospitalization according to DSM-5 criteria (American Psychiatric Association & American Psychiatric [34]). Catatonia being a transnosographic condition [35], we selected catatonia patients in two different disorders, namely schizophrenia or schizoaffective disorder. Therefore, we selected catatonia patients with (1) a diagnosis of schizophrenia or schizoaffective disorder and (2) underwent an anatomical T1 MRI sequence. For each patient, we collected the following demographic and clinical information: age, sex, duration of hospitalization, associated psychiatric or nonpsychiatric pathology, age at onset of schizophrenia or schizoaffective disorder, total score on the BFCRS (if available) at the time of diagnosis and when electroconvulsive therapy (ECT) was performed, the number of ECT sessions.

We then selected schizophrenia patients without catatonia, group-matched for age, sex, underlying psychiatric pathology, and age of onset. We therefore retrospectively searched for all inpatients in the university hospital department at Sainte-Anne Hospital, Paris, France, between January 2015 and March 2022 with (1) a diagnosis of schizophrenia or schizoaffective disorder and (2) who underwent an anatomical T1 MRI sequence. The presence of catatonic episode according to DSM-5 criteria and ultra-resistant schizophrenia, that is, persistence of symptoms despite well-conducted clozapine treatment, were exclusion criteria. For each patient, we collected the following demographic and clinical information: age, sex, duration of hospitalization, age at onset of schizophrenia or schizoaffective disorder, and when ECT was performed, the number of ECT sessions.

Schizophrenia patients, with or without catatonia, were compared with 82 healthy controls with (1) no psychiatric history and (2) available anatomical T1 MRI sequence.

**Figure 1.**
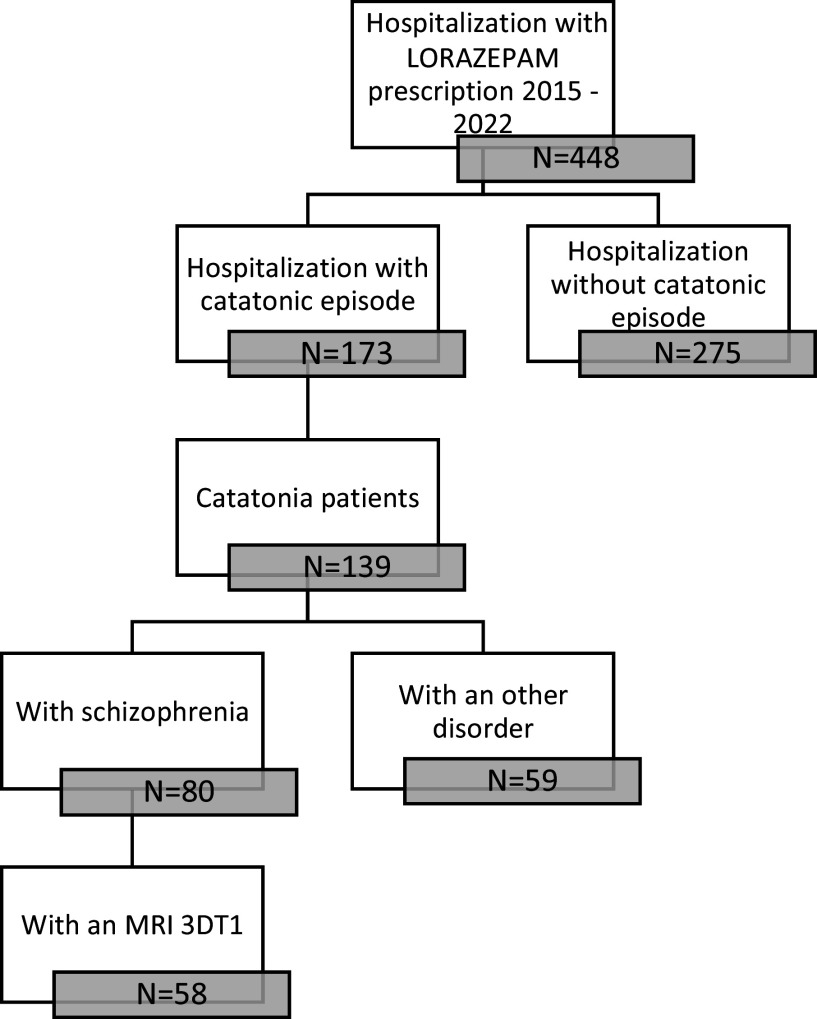
Flow chart of the retrospective selection of the patients.

Demographic and clinical details of the study samples are reported in Table 1. The flow chart of patient selection is reported in Figure 1.

**Table 1.** Study population description

	SSD-c (*N* = 58)	SSD-nc (*N* = 65)	HC (*N* = 82)	SSD-c versus SSD-nc (*p-*value)^a^
Men: Women	40:18	44:21	39:43	1
Sex ratio (Men/Women)	2.2	2.1	0.91	1
Age, M ± SD years	36 ± 17	37 ± 13	29 ± 8	0.4
Schizophrenia, *N* (%)	48 (83)	53 (82)	–	1
Schizoaffective disorder, *N* (%)	10 (17)	12 (18)	–	1
Age at onset, M ± SD years	25.1 ± 9.8	28.3 ± 10.3	–	.07
Average length of hospitalization, M ± SD days	76.3 ± 61	54.2 ± 51.2	–	**.02**
ECT, *N* (%)	30 (52)	6 (9)	–	**<.0001**
ECT, M ± SD session numbers	12.5 ± 7.4	13 ± 7.7	–	0.99
Assessment of DSM-5 criteria for catatonia	58 (100)	65 (100)		
BFCRS evaluation, *N* (%)	27 (47)	–	–	–
BFCRS score, M ± SD days	17 ± 7	–	–	–
1,5 T GE/3 T GE/3 T CANON, *N* (%)	50 (86)/8 (14)/0 (0)	54 (83)/11 (17)/0 (0)	0 (0)/20 (24)/62 (76)	0.96

*Note:* Bold font corresponds to significant effects.

Abbreviations: HC, healthy controls; SSD, schizophrenia; SSD-c, schizophrenia patients with catatonia; SSD-nc, schizophrenia patients without catatonia.

a chi-squared test is used for comparison.

This study was authorized by the data protection delegation of the GHU-Paris Psychiatry and Neurosciences under reference number D22-R003 as part of the retrospective trials on data covered by reference methodology MR004.

### MRI acquisition

Anatomical T1 MRI were acquired on the MRI scanners at the brain imaging facility of Sainte-Anne hospital (CEREN), 3 T Canon and General Electric and 1,5 T General Electric (see Table 1). Of note, previous studies have shown that the classification of the OFC sulcal pattern is not influenced by the scanner nor the acquisition sequence [25, 36]. Four participants (one schizophrenia patient with catatonia, two schizophrenia patients without catatonia, and one healthy control) were excluded because of motion artifacts leading to blurred OFC region. In addition, two schizophrenia patients without catatonia were also excluded because of the presence of frontal meningiomas.

### MRI analysis

We followed the same procedure as in previous studies of OFC sulcal patterns [18, 21, 22, 25, 27, 37]: (1) the anatomical MRI were linearly spatially normalized on a MNI template and aligned along the anterior commissure-posterior commissure plan using volBrain (http://volbrain.upv.es) [38]; (2) all images were then resampled to a common 1 × 1 × 1 mm^3^ voxel resolution and a volume size of 181 × 217 × 181 voxels; and (3) intensity were normalized after inhomogeneity correction.

### Regional brain volumes comparison

To ensure comparability between the two groups of patients in terms of the severity of the underlying pathology, we opted to evaluate regional brain volumes since we did not have access to clinical scales such as the Positive and Negative Syndrome Scale (PANSS). These brain volumes were previously identified as an indirect measure of schizophrenia severity, including the gyrus rectus, superior temporal gyrus ([39, 40]), the lateral ventricle and caudate volume [41]. Insula and anterior cingulate cortex (ACC) volumes were not included in the analysis as they show decreased volume in catatonia patients with schizophrenia [42], making it impossible to disentangle the effects of schizophrenia from that of catatonia. Regional brain volumes were obtain using VolBrain [38]. The analyses did not reveal any significant differences in regional brain volume between the two patient groups, which suggest that patients with or without catatonia similar have similar clinical severity (see Supplementary Table S1). Additionally, we included a measurement of overall brain volume, which has been shown to have a correlation with the sulcation of the prefrontal cortex [43] in order to rule out any potential influence of brain size on the observed differences in sulcal patterns (see Supplementary Table S1).

### OFC sulcal pattern classification

**Figure 2.**
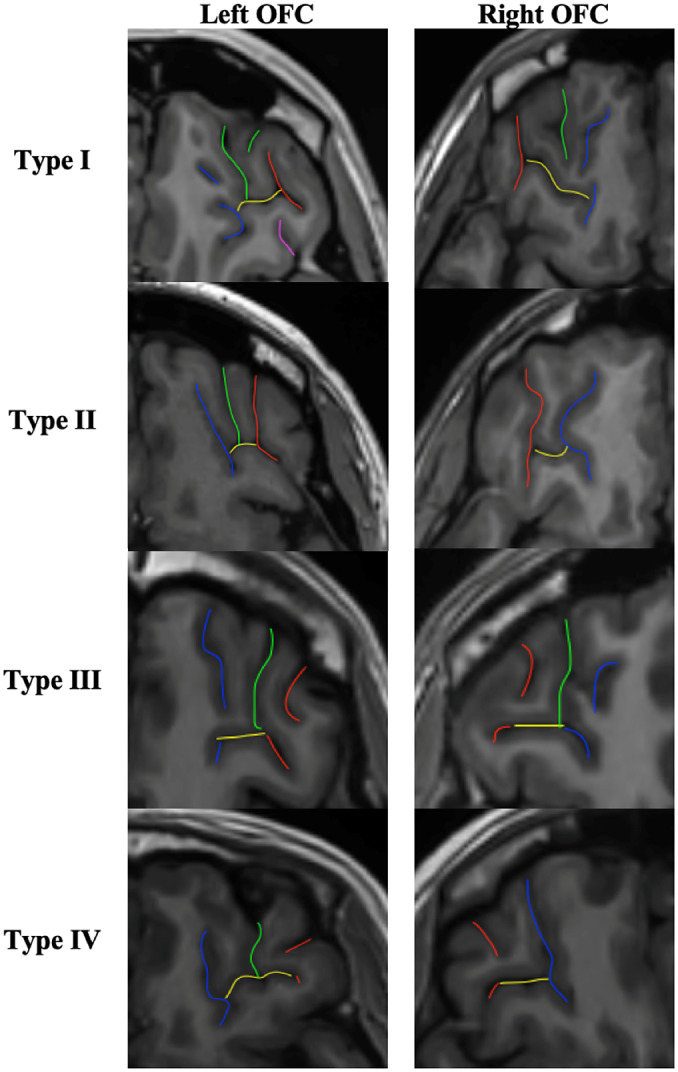
OFC sulcal pattern classification. Red sulci correspond to the lateral orbital sulcus (LOS), blue sulci to the medial orbital sulcus (MOS), yellow sulci to the transverse orbital sulcus (TOS), green and purple sulci to the intermediate orbital sulcus (IOS) and posterior orbital sulcus (POS) respectively. In type I, the rostral and caudal portions of the MOS are disconnected whereas the rostral and caudal portion of the LOS are connected. In type II, the rostral and caudal proportions of both MOS and LOS are connected and joined together by the transverse orbital sulcus (TOS). In type III, the rostral and caudal proportions of both MOS and LOS are disconnected. In the rare type IV, subgroup of type III, the rostral and caudal portions of the LOS are disconnected whereas the MOS continues. Additional sulci of the OFC were also identified, including the intermediate orbital sulcus (IOS) and the posterior orbital sulcus (POS). Additional sulci anterior to the TOS are IOS and posterior to the TOS are POS. Note that the IOS and POS can be dual (medial and lateral IOS and POS).

The classification of OFC sulcal pattern followed the standard visual inspection procedure of Chiavaras and Petrides [17], based on the continuity of the medial orbital sulcus (MOS) and lateral orbital sulcus (LOS) (see Figure 2).

The same procedure as previously described [23, 25] was used:

1. On the axial section: identify the olfactory sulcus and with the slice intersection cross, slide it along the olfactory sulcus in order to find in the coronal section the good view of the sulci.
2. On the coronal section: choose the best slice to identify the MOS LOS and intermediate sulci. MOS is the closest to the OS except if the sulcus <3.75 mm. In this case, it is a sulcus fragmentosus (Fr). LOS is the most lateral sulcus that stays in the OFC region and under the ACPC line.
3. Once the MOS LOS are identified, draw a line in the MOS and the LOS on the coronal section in order to highlight the MOS and LOS in the axial view. On the axial view, identify the MOS and LOS thanks to the markup.
4. On the axial view, identify the TOS. Take the lowest slice where you still see the TOS. And draw the TOS on the axial view. Check the TOS on the sagittal view thanks to the slice intersection cross.
5. Once the MOS LOS and TOS are identified on the axial view, determine if the rostral and caudal portion of both the MOS and LOS are connected or disconnected. Here the LOS is connected and the MOS is disconnected. Check the connection between the rostral and caudal portion of the MOS and LOS on the coronal view.
6. Then identify the IOS and POS: On the axial view, put the slice intersection cross on the TOS level and front and back on the coronal view. All the supplementary sulci, between the MOS and the LOS, in front of the TOS are IOS and all the supplementary sulci, between the MOS and the LOS, back of the TOS are POS.

The 3D slicer software (http://www.slicer.org) was used for visual inspection of the OFC sulcal pattern as in previous studies [21, 23, 27, 37].

All MRI data were anonymized. The two hemispheres have been classified independently (M.M.). Classification of the sulcal pattern type was blind to the diagnoses and the sulcal pattern in the contralateral hemisphere. An independent rater (M.P.) independently classified the sulcal pattern type for 50 randomly selected hemispheres. The intraclass correlation coefficient (Cronbach’s alpha) for OFC sulcal pattern was 0.91 for the left hemisphere and 0.93 for the right hemisphere. If the two experts disagree in the classification of the sulcus morphology of a given participant, a third expert joined the two other experts to reach a consensus.

### Statistical analysis

Multinomial logistic regressions were used to compare the distribution of OFC sulcal pattern (“Type I” vs. “Type II” vs. “Type III”) in the left and right hemispheres with group (“schizophrenia with catatonia” vs. “schizophrenia without catatonia” vs. “healthy controls”) and gender (“male” vs. “female”) as categorical factor and age as continuous covariate. Post hoc comparisons, with Tukey correction for multiple tests, were used to investigate distribution differences between pairs of subgroups.

We then examined the extent to which OFC sulcal pattern was associated with clinical features of patients with catatonia, including age of onset of the psychiatric disease, ECT treatment (“yes” vs. “no”), number of ECT sessions, duration of hospitalization and BFCRS score, using linear and logistic regression models.

Medications measures were not entered in the analyses since medication have no effect on OFC sulcal patterns [23, 44].

All statistical analyses were performed using the R 4.0.1 software (http://www.r-project.org) running on Rstudio with “nnet,” “multcomp,” “car,” “effects,” and “lsmeans” libraries [45–46].

## Results

### Between-group comparisons

**Figure 3.**
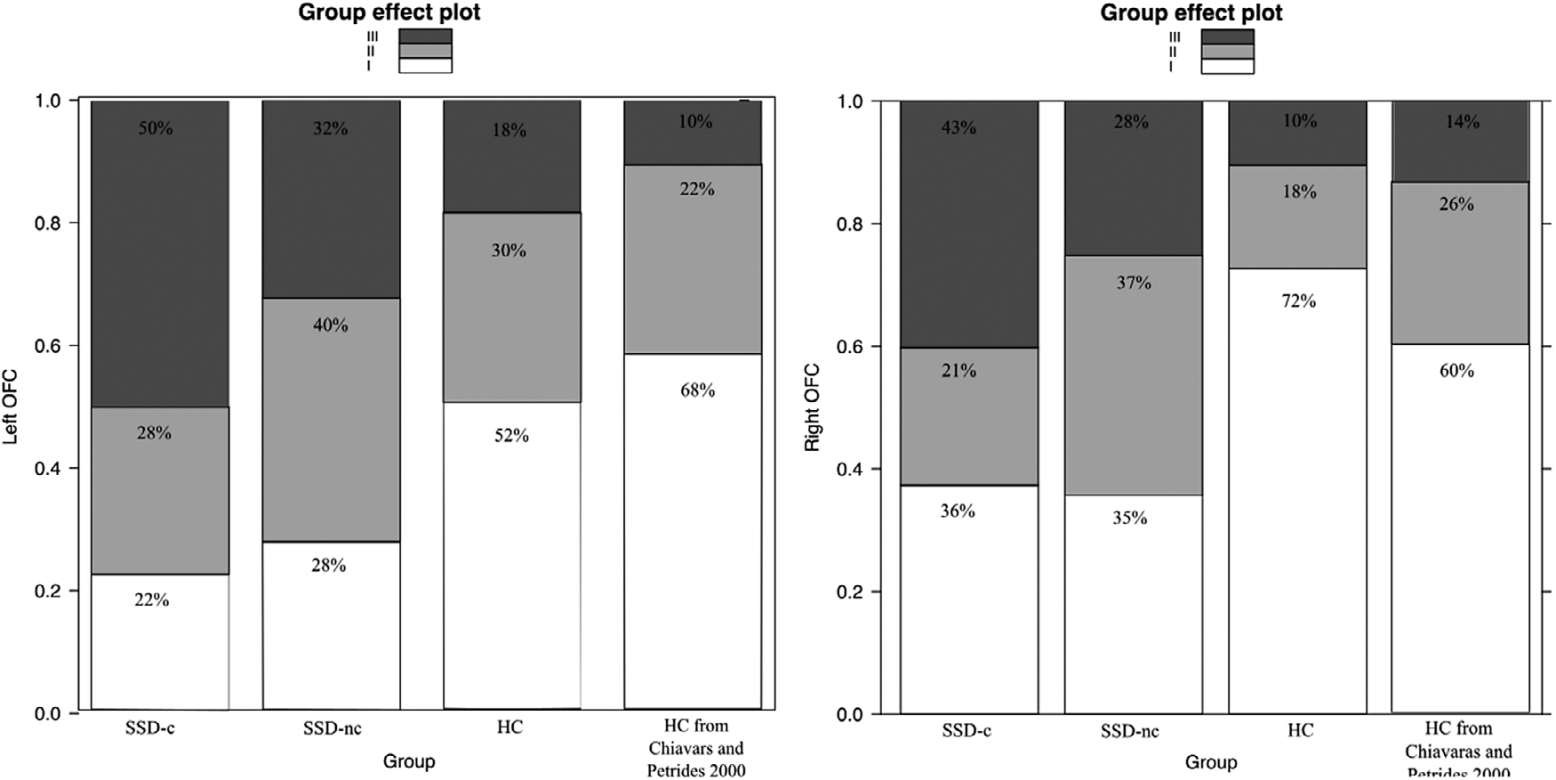
OFC sulcal pattern distribution. HC, healthy control; SSD, schizophrenia; SSD-c, schizophrenia patients with catatonia; SSD-nc, schizophrenia patients without catatonia.

Of the 410 classified hemispheres, we found 176 (43%) type I, 118 (29%) type II, and 116 (28%) type III, including 16 (4%) type IV, a rare subgroup of type III. The distribution by group and by hemisphere is reported in Table 2 and Figure 3.

**Table 2.** OFC sulcal pattern distribution

OFC	SSD-c (*N* = 58)	SSD-nc (*N* = 65)	HC (*N* = 82)	Main effect of groups (*p-*value)	Intergroup comparison *p-*value		
					SSD-c versus HC	SSD-c versus SSD-nc	SSD-nc versus HC
Left							
Type I	13 (22)	18 (28)	42 (51)	**.001**	**.0005**	0.43	**.05**
Type II	16 (28)	26 (40)	25 (30)		0.78	0.11	0.57
Type III	29 (50)	21 (32)	15 (18)		**<.0001**	**.02**	0.22
Right							
Type I	21 (36)	23 (35)	59 (72)	**1.1 e-05**	**<.0001**	0.93	**.003**
Type II	12 (21)	24 (37)	15 (18)		0.34	**.04**	**.05**
Type III	25 (43)	18 (28)	8 (10)		**<.0001**	**.05**	0.12

*Note: Data are reported as N (%). Bold font corresponds to significant effects.*

Abbreviations: HC, healthy controls; SSD, schizophrenia; SSD-c, schizophrenia patients with catatonia; SSD-nc, schizophrenia patients without catatonia.

In the healthy group, the proportion of the three OFC sulcal pattern types was similar in the left (*χ* ^2^ = 3.7493, *p* = 0.1534) and the right (*χ* ^2^ = 2.0201, *p* = 0.3642) hemisphere, to the seminal study by Chiavaras and Petrides [17].

The multinomial regression model showed a main effect of group on OFC sulcal pattern in the left (*χ* ^2^ = 18.1607; *p* = .001) and right (*χ* ^2^ = 28.2945; *p* = 1.08e-05) hemispheres. Sex and age had no effect on left and right OFC sulcal pattern (all *p*s > 0.4).

Post hoc pairwise comparisons revealed that catatonia patients had more type III on the left (*t*-ratio = 8.95; *p* < .0001) and the right (*t*-ratio = 9.578; *p* < .0001) OFC compared to healthy controls. Similarly, catatonia patients had more type III on the left (*t*-ratio = 3.189; *p* = .02) and the right (*t*-ratio = 2.738; *p* = .05) OFC compared to schizophrenia patients without catatonia. In addition, catatonia patients had less type I on the left (*t*-ratio = −5.785; *p* = .0005) and the right OFC (*t*-ratio = −8.740; *p* < .0001) compared to healthy controls and had less type II compared to schizophrenia patients without catatonia patients in the right OFC (*t*-ratio = −2.907; *p* = .04).

Schizophrenia patients without catatonia had less type I in the right (*t*-ratio = −4.470; *p* = .003) and the left (*t*-ratio = −2.720; *p* = .05) and more type II in the right OFC (*t*-ratio = 2.738; *p* = .0502) compared to healthy controls.

All details on the OFC sulcal pattern distribution and intergroup comparisons can be found in Table 2 and Figure 3.

Analysis of the distribution of IOS and POS sulcal pattern can be found in Supplementary Table S2.

### Clinical correlations

Analysis of clinical correlations within the subgroup of catatonia patients revealed a significant main effect of the left OFC sulcal pattern on the BFCRS score (*F* = 423.93; *p* = .02). BFCRS score was found to be higher in type III than in type II (*t*-ratio = −3.105; *p* = .02); there was no BFCRS score difference between the left OFC type I and type II (*t*-ratio = −1.508; *p* = 0.31), and between type I and type III (*t*-ratio = −1,009; *p* = 0.58). The left OFC sulcal pattern also had an effect on the number of ECT sessions (*F* = 4.3610; *p* = .027), the type I being associated with more ECT sessions compared to type II (*t*-ratio = 2.951; *p* = .020). There was no difference in the number of ECT sessions between type I and type III (*t*-ratio = 2.176; *p* = 0.10), and between type II and type III (*t*-ratio = −1,652; *p* = 0.25). The right OFC sulcal pattern had an effect on the duration of hospitalization (*F* = 3.7082; *p* = .03), the type II being associated with longer hospitalization compared to the type I (*t*-ratio = −2.503, *p* = .04) and the type III (*t*-ratio = 2.444; *p* = .046).

There was no effect of the OFC sulcal pattern on the age of onset of psychiatric illness nor ECT treatment nor the total number of catatonic episodes (all *p*s > 0.29).

Details of the clinical correlations can be found in Table 3.

**Table 3.** Correlation between clinical data and OFC sulcal pattern in catatonia patient subgroup

		OFC	SSD-c (*N* = 58)	Main effect of type (*p*-value)	Intergroup comparison *t*-ratio (*p-*value)		
					I versus II	I versus III	II versus III
Age at onset, M ± SD years	Left OFC	I	24.9 ± 9.5	0.89			
		II	24.7 ± 9.6				
		III	25.1 ± 9.8				
	Right OFC	I	24.8 ± 9.6	0.52			
		II	24.5 ± 9.7				
		III	25.5 ± 9.8				
ECT, *N* (%)	Left OFC	I	5 (38)	0. 29			
		II	7(44)				
		III	19(66)				
	Right OFC	I	10(48)	0.64			
		II	8(66)				
		III	13(52)				
ECT, M ± SD number sessions	Left OFC	I	20 ± 7.3	**.03**	**2.95 (.020)**	2.18 (.099)	−1.6 (0.25)
		II	8.3 ± 7.2				
		III	11.8 ± 7.4				
	Right OFC	I	8.6 ± 7.4	.089			
		II	15.8 ± 7.4				
		III	12.8 ± 7.5				
BFCRS score, M ± SD	Left OFC	I	18 ± 7.6	.02	1.51 (0.31)	−1.01 (0.58)	**−3.10 (.02)**
		II	13.4 ± 6.9				
		III	23 ± 7.6				
	Right OFC	I	16.1 ± 6.6	0.25			
		II	21.7 ± 7.4				
		III	20.1 ± 7.6				
		II	17.7 ± 7				
		III	18.3 ± 7.7				
Hospitalization duration, M ± SD days	Left OFC	I	80.6 ± 70.2	0.58			
		II	84.7 ± 70.4				
		III	80.53 ± 68.8				
	Right OFC	I	80.8 ± 69.4	**.03**	**−2.50 (.04)**	−0.17 (0.98)	**2.44 (.04)**
		II	90 ± 72				
		III	81.4 ± 71.1				
Number of catatonic episodes M ± SD	Left OFC	I II III	1.45 ± 0.8 1.47 ± 0.8 1.48 ± 0.9	0.95			
	Right OFC	I	1.49 ± 0.9	0.43			
		II	1.46 ± 0.9				
		III	1.41 ± 0.8				

*Note:* Bold font corresponds to significant effects.

Abbreviations: HC, healthy controls; SSD, schizophrenia; SSD-c, schizophrenia patients with catatonia; SSD-nc, schizophrenia patients without catatonia.

## Discussion

In this first study that investigated the OFC sulcal pattern in catatonia, we found a specific OFC sulcal pattern distribution in schizophrenia patients with catatonia in comparison with healthy controls but also with schizophrenia patients without catatonia. Catatonia patients were found to have more type III OFC pattern than schizophrenia patients without catatonia and healthy subjects, less type I than healthy subjects, and less type II than schizophrenia patients without catatonia.

The increased type III in schizophrenia patients with catatonia is in line with previous studies showing more type III in schizophrenia patients, including in early stages of the illness [24], and specifically in patients with more severe forms of schizophrenia [23, 27]. Indeed, catatonia can be considered a clinical complication of schizophrenia, which burdens the prognosis of patients with schizophrenia [30]. Our findings therefore support that type III is associated with poor prognosis in schizophrenia. Furthermore, type III could also predict the severity of the catatonic episode, a high BFCRS score being associated with a type III organization. In addition, OFC sulcal patterns could be used as therapeutic predictive marker. Indeed, left OFC sulcal patterns appear to be associated with the number of ECT sessions in schizophrenia patients with catatonia, conferring possible resistance to ECT in a particular subtype of left sulcal patterns. Hence, the type III sulcal pattern could potentially be a marker of the severity of the pathology, intimately linked to its neurodevelopmental load [23].

The OFC is divided into several subregions that have distinct cellular architecture and connectivity [49]. These segments are densely connected with subcortical and cortical regions. Increased type III OFC have been shown to be associated with abnormal white matter connectivity in the uncinate fasciculus connecting the OFC to limbic regions in bipolar patients [50]. In catatonia, several authors have hypothesized impaired connectivity of networks including the OFC, limbic regions, and cortico-subcortical loops involved in cognitive, emotional, and motor processing [7, 10, 51]. Such hypothesis was supported by a diffusion MRI study in catatonia revealing abnormal microstructure in the white matter bundles connecting orbitofrontal with parietal, thalamic, and striatal regions [52, 53]. We speculate that type III OFC pattern in catatonia patients may be associated with abnormal connectivity of the uncinate fasciculus connecting the OFC to limbic regions with negative emotional overload related to the lack of inhibitory feedback from the OFC to the limbic system. Indeed, catatonia is viewed by several authors as a primitive reaction to fear with the onset of stupor in response to threat [54], catatonia patients experiencing fear during their catatonic episode [55, 56]. The limbic system has been shown to be altered in catatonia patients at the structural level [57] and also at the functional level during the processing of negative emotions [11, 13]. Further studies should investigate the possible link between such abnormal anatomo-functional dysconnectivity and abnormal OFC sulcal patterns.

The OFC type III may be a trait marker of neurodevelopmental vulnerability to the catatonic syndrome. Indeed, the OFC sulcal pattern is determined in utero, between 28 and 31 weeks of gestation [58, 59]. Previous research has reported postnatal stability of the sulcal pattern among various cortical regions, including the occipito-temporal cortex and various areas of the prefrontal cortex, such as the anterior cingulate cortex and the inferior frontal sulcus [60]. Of note, the stability after birth of the OFC sulcal pattern has yet to be investigated. Unlike the quantitative features of the cortical sheet such as thickness, surface area, or curvature), which take decades to reach the levels observed in adult, the qualitative sulcal patterns of postnatal cortex anatomy serve as a proxy for earlier developmental events [61]. Moreover, several studies have reported increased type II [62] and type III [18] associated with prematurity. OFC sulcal pattern is likely more influenced by the fetal environment than by genetic factors since a recent study found a low concordance rate of OFC sulcal patterns between monozygotic and dizygotic twin pairs [63]. It would be worthwhile to explore longitudinal data on later neurodevelopmental elements in addition to those in early stages so as to trace the trajectory of neurodevelopment.

Several methodological issues call for caution when interpreting our results. First, this study is based on a retrospective analysis of clinical data. We used a rigorous approach to determine the history of catatonia from clinical records by assessing the presence of DSM-5 diagnostic criteria in both groups as well as treatment response. However, as discussed in a previous retrospective study [64], assessment of catatonia criteria is not always straightforward because of under-recognition of the syndrome [65]. Furthermore, it is possible for patients classified in the noncatatonia group to develop catatonia in the future, despite our 2 to 8 years follow-up after patient assessment. Additionally, no other clinical scales were available to assess the severity of the underlying disease. The regional brain volume analyses did not reveal any differences in brain volume between the two groups of patients, indicating similar clinical severity in patients with or without catatonia (see Supplementary Table S1). Of note, it is difficult to completely rule out the possibility that the type III sulcal pattern may be linked to another behavioral dimension. Second, the data were acquired on the same site but with different MRI scanners. It is important to note that previous studies revealed the MRI scanner does not influence the OFC sulcal pattern classification [25, 36], mainly because the sulcal pattern is a stable gross anatomical cortical feature, largely independent from the MRI signal and voxel size [66]. In order to control for possible effect of the MRI field strength, we carefully matched the subgroups of schizophrenia patients with catatonia and schizophrenia patients without catatonia. In addition, we reran the statistical models with the MRI field as cofounding factors and the results remained very similar. Third, healthy control subjects were not matched on age and sex with the two patient groups. Similarly, age and sex were therefore added in the statistical analyses as possible confounders. Of note, we used type III analysis of variance in the statistical analyses because they are robust to such unbalanced designs. Fourth, because ECT is part of the recommended treatment for catatonia [31, 32], schizophrenia patients with catatonia received significantly more ECT treatment compared to schizophrenia patients without catatonia. ECT can induce local changes of cortical volume [67], however, it could unlikely affect sulcal patterns which are nonplastic, very stable [61, 68], markers of the cortex anatomy. Fifth, we used the BFCRS, the current gold standard scale for assessing catatonia and its severity [33]. The OFC is involved in emotional processing [6] and the Northoff catatonia scale [69], which evaluates the affective dimension of the catatonic syndrome, could also be of interest. Finally, it is worth noting that catatonia is a transnosographic syndrome. The findings from this study, which focused on patients with schizophrenia or schizoaffective disorders, should be confirmed and expanded toward other psychiatric conditions, including autism spectrum disorder as well as medical conditions like encephalitis [70]. A transdiagnostic approach [24, 25] is necessary to confirm whether type III OFC sulcal patterns are markers of catatonia, or if they are exclusively specific markers of catatonia in psychosis. In addition, this study focuses on the OFC, the sulcal patterns of other brain regions likely involved in the complex pathophysiology of catatonia [8] should be explored in further studies.

In conclusion, this is the first study to provide evidence of abnormal OFC sulcal patterns in schizophrenia patients with catatonia, with more type III than in healthy subjects and in schizophrenia patients without catatonia, supporting a neurodevelopmental component of catatonia, at least in schizophrenia patients. Catatonia neurodevelopmental component is increasingly recognized and needs to be further investigated notably in nonpsychosis catatonia patients. Such investigations aim to improve patient characterization and explore the underlying pathophysiological mechanisms of catatonia.

## Supporting information

Moyal et al. supplementary material Moyal et al. supplementary material

## Supplementary material

The supplementary material for this article can be found at https://doi.org/10.1192/j.eurpsy.2023.2461.
