# Supplementary material for: Orbitofrontal sulcal patterns in catatonia
Source: Eur Psychiatry. 2023 Oct 19;67(1):e6. doi: 10.1192/j.eurpsy.2023.2461 (PMC10964273; doi:10.1192/j.eurpsy.2023.2461)
Supplement: Moyal et al. supplementary material [file S0924933823024616sup001.docx]

**SUPPLEMENTARY MATERIALS**

**Regional brain volume comparisons**

**Table S1. Regional brain volumes.** Abbreviations: SSD=schizophrenia; SSD-c=Schizophrenia patients with catatonia; SSD-nc=Schizophrenia patients without catatonia, HC=Healthy Controls. *Normalized by intracranial volume, **T-tests were used for comparison between groups.

|  | SSD-c  (N=58) | SSD-nc  (N=65) | HC  (N=82) | SSD-c  vs  SSD-nc (p value) ** |
| --- | --- | --- | --- | --- |
| Intracranial volume cm3 | 1361.9±141.1 | 1397.3±129.6 | / | 0.15 |
| Gray matter volume* % | 50.1±3.4 | 49.1±3.6 | / | 0.11 |
| Cortical gray matter volume* % | 39.2±3.0 | 38.3±3.0 | / | 0.08 |
| Subcortical gray matter volume* % | 3.04±0.3 | 3.01±0.3 | / | 0.59 |
| Superior temporal gyrus total volume* % | 1.03±0.1 | 1.04±0.1 | / | 0.50 |
| Lateral ventricle total volume* % | 1.69±1.1 | 1.42±0.7 | / | 0.11 |
| Gyrus rectus total volume* % | 0.30±0.04 | 0.30±0.04 | / | 0.48 |
| Caudate total volume* % | 0.53±0.1 | 0.53±0.1 | / | 0.66 |

**Difference of IOS and POS distribution between groups**

We found an IOS in 340 (83%) hemispheres and a POS in 128 (31%) hemispheres. Statistical analyses revealed a main effect of group on the number of IOS (F=5.9692; p=.003) and number of POS (F=4.1795; p=.02) in the right hemisphere, but not in the left hemisphere (all ps > 0.16) (see **Table S2**).

Post-hoc pairwise comparisons further revealed that schizophrenia patients without catatonia had significantly less IOS (t ratio=-3.364; p=.003) and POS (t ratio=-2.731; p=.02) in the right hemisphere compared to healthy subjects. There was a trend toward less POS (t ratio=-2.190; p=.07) in the right hemisphere and more IOS (t ratio=2.299; p=.06) in catatonia patients compared to schizophrenia patients without catatonia (see **Table S2**).

The increased number of sulci found in schizophrenia patients with catatonia could be related to the hypergyrification reported in catatonia (Hirjak et al. 2019; Walther et al. 2022).

**Table S2. IOS and POS distribution.** Data are reported as N (%). Abbreviations: SSD=Schizophrenia; SSD-c=Schizophrenia patients with catatonia; SSD-nc=Schizophrenia patients without catatonia, HC=Healthy Controls. Bold font corresponds to significant effects.

| **OFC** | **SSD-c**  **(N=58)** | **SSD-nc**  **(N=65)** | **HC**  **(N=82)** | **Main effect of groups**  (p value) | **Inter-group comparison**  p value | | |
| --- | --- | --- | --- | --- | --- | --- | --- |
|  |  |  |  |  | SSD-c vs HC | SZ-c vs SZ-nc | SSD-c vs HC |
| **Left** |  |  |  |  |  |  |  |
| IOS | 53 (91) | 47 (72) | 66 (80) | 0.17 | - | - | - |
| POS | 17 (29) | 17 (26) | 32 (39) | 0.18 | - | - | - |
| **Right** |  |  |  |  |  |  |  |
| IOS | 51 (88) | 50 (77) | 74 (90) | .003 | 0.57 | .05 | **.003** |
| POS | 14 (24) | 13 (20) | 34 (14) | .01666 | .07 | 0.88 | **.02** |
